# Physicochemical, Sensory, and Microbiological Analysis of Fermented Drinks Made from White Kidney Bean Extract and Cow’s Milk Blends during Refrigerated Storage

**DOI:** 10.3390/microorganisms12091832

**Published:** 2024-09-04

**Authors:** Ibaratkan Kurbanova, Lina Lauciene, Kristina Kondrotiene, Gintare Zakariene, Vitalijs Radenkovs, Sandra Kiselioviene, Alvija Salaseviciene, Agne Vasiliauskaite, Mindaugas Malakauskas, Mukarama Musulmanova, Loreta Serniene

**Affiliations:** 1 Department of Food Production Technology, Kyrgyz State Technical University Named after I. Razzakov, 66, Chyngyz Aitmatov Ave, Bishkek 720044, Kyrgyzstan; ibaratk@gmail.com (I.K.);; 2 Department of Food Safety and Quality, Veterinary Academy, Lithuanian University of Health Sciences, Tilzes Str. 18, LT-47181 Kaunas, Lithuanialoreta.serniene@lsmu.lt (L.S.); 3 Research Laboratory of Biotechnology, Division of Smart Technologies, Latvia University of Life Sciences and Technologies, Riga Str. 22B, LV-3004 Jelgava, Latvia; 4 Institute of Horticulture (LatHort), LV-3701 Dobele, Latvia; 5 Food Institute, Kaunas University of Technology, Radvilenu Str. 19, LT-44239 Kaunas, Lithuania

**Keywords:** white kidney bean extract, cow’s milk, blends, fermentation, probiotic-type strains

## Abstract

Due to its low dietary impact and bioactive compounds, such as polyphenols and flavonoids, white kidney bean extract is an attractive raw material for fermented drinks. It can be utilized either on its own or blended with cow’s milk, offering a promising solution to help meet dairy product demand during mid-season shortages. Therefore, this study aimed to explore the physicochemical characteristics, sensory properties, and microbiological profile of fermented milk-like drinks made from white kidney bean extract, cow’s milk and their blends during 28 days of storage at 4 °C. Three blends of fermented milk-like drinks (FMLDs) were prepared from different ratios of cow’s milk (CM) and kidney bean extract (BE): FMLD1 (CM 30%:BE 70%); FMLD2 (CM 50%:BE 50%), FMLD3 (CM 70%:BE 30%), along with plain fermented kidney been extract (FBE; CM 0%:BE 100%), and plain fermented cow’s milk (FCM; CM 100%:BE 0%). The mixtures were pasteurized at 92 °C for 25 min and fermented with a probiotic-type starter culture (*S. thermophilus*, *B. bifidum*, *L. acidophilus*) at 43 °C. FBE exhibited the lowest levels of carbohydrates (2.14%), fat (0.11%), and protein (1.45%) compared to fermented cow’s milk and blends. The FBE and the fermented blends with a higher ratio of bean extract had lower viscosity and lactic acid contents, greener hue, more pronounced aftertaste and off-flavors, and received lower overall acceptability scores. Although the FCM had higher counts of *S. thermophilus* and *L. acidophilus*, the FBE displayed significantly higher counts of *B. bifidum*. This study demonstrated the potential of using white kidney bean extract and its blends with cow’s milk to create unique fermented products with a lower dietary impact, highlighting the importance of further optimizing the formulations to enhance sensory qualities and reduce the beany off-flavors in the products with added kidney bean extract.

## 1. Introduction

Kidney beans (*Phaseolus vulgaris* L.) belong to the legume family (*Fabaceae*) and contain high levels of dietary fiber (29.32–46.77%), resistant starch (9.16–18.09%), and protein (22.06–32.63%), but low levels of lipids (1.05–2.83%) and sugars (1.55–9.07%) [1]. Important compounds present in beans include phenolic compounds, tocopherols, peptides, amino acids, unsaturated fatty acids, and minerals such as calcium (3 g/kg), iron (40 mg/kg), and zinc (35 mg/kg), which can provide various biological activities [2]. According to Wang et al. (2020) [3], *Phaseolus vulgaris* extract (PVE) has potential effects on human health and possesses antioxidant, anti-carcinogenic, anti-inflammatory, anti-obesity, anti-diabetes, and cardioprotective properties.

Kyrgyz kidney beans have emerged as a relatively recent crop in Kyrgyzstan, marking a shift from the historical cultivation of other legumes such as peas and soybeans. Since the late 1990s, there has been a notable rise in kidney bean production, driven by FBE and an export-oriented approach, showcasing the sustained dominance of kidney bean cultivation in this region [4]. Despite the high export potential of beans and their role in the employment sector in an entire region of the Kyrgyz Republic, domestic consumption of beans in the country remains at a low level, and there is no processing of beans into high nutritional food. The local population does not benefit from the kidney bean’s high protein content and valuable nutritional properties [5]. At the same time, plant-based/non-dairy beverages are in demand in the world due to increased cow’s milk allergies, lactose intolerance, calorie problems, and hypercholesterolemia. Many consumers demand plant-based milk substitutes for healthier diets, lifestyles, or vegan foods. Moreover, they are inexpensive milk alternatives and can be used in the countries and places where cow’s milk is scarce [5].

Increasing the share of plant-based foods in diets can offer several sustainability benefits. Plant-based foods generally have a lower environmental footprint compared to dairy products in terms of land and water use, greenhouse gas emissions, and energy consumption. Shifting towards a more plant-based diet can help alleviate pressure on natural resources and reduce the environmental impact of food production [6]. As mentioned earlier, blended food compositions can help meet the demand for dairy products during mid-seasons, when many countries experience shortages in milk deliveries. Given that white kidney beans are the most common, accessible, and preferred crop among the population of Kyrgyzstan, they were selected for the production of milk-like drinks in our research. The water-based, milk-resembling drinks were used either alone or blended with cow’s milk in various proportions to create fermented beverage options. The suitability of such drinks to partially replace cow’s milk during periods of scarcity has not been investigated before.

Traditional milk yoghurt is an accepted and widely consumed dairy product due to its rich composition and essential properties. However, the growing popularity of plant-based yoghurt-like drinks can indeed be attributed to its unique nutritional composition and several other factors, as plant-based milk alternatives offer a diverse range of bioactive components, such as phenolic compounds and flavonoids, which are known for their antioxidant properties. Additionally, many plant-based beverages are naturally low in fat and carbohydrates, making them appealing options for those looking to manage their weight or dietary intake. The increasing adoption of vegetarian, vegan, and flexitarian diets has fueled the demand for plant-based milk alternatives. These dietary lifestyles are often motivated by ethical, health, or environmental concerns, and plant-based milks align well with these principles, providing a cruelty-free, sustainable option for consumers [7].

Despite its benefits, kidney beans present challenges due to phytohaemagglutinin, a natural toxin in raw kidney beans that can cause gastrointestinal distress if not properly processed [8]. Ensuring safe production is essential for consumer safety. Additionally, the flavor and texture of kidney bean extract may not appeal to all consumers [9]. Ayivi et al. (2022) [10] state that fermentation can improve the taste and nutritional properties, contributing to a thicker consistency and smoother texture due to the production of polysaccharides and other metabolites during fermentation. Moreover, probiotic-type strains, present in the starter, can influence the texture and consistency of fermented drinks. They can outcompete spoilage microorganisms, thus enhancing safety and potentially extending the shelf life of the product. Additionally, incorporating probiotics introduces live beneficial bacteria, enhancing the health-promoting properties of fermented products [10].

This study aimed to explore the physicochemical, sensory, and microbiological characteristics of fermented milk-like drinks made from white kidney bean extract, cow’s milk, and their blends. This investigation spanned 28 days of storage at 4 °C, with assessments conducted at 7-day intervals to understand the effects on these diverse milk compositions. 

## 2. Materials and Methods

### 2.1. Raw Materials

The dried matured white kidney beans (*Phaseolus vulgaris* L.) were obtained from the Talas market in Kyrgyzstan (harvested in 2023; 18.2% moisture, 18.0% protein, 1.5% fat, 53.9% carbohydrate, 6.6% crude fiber). Pasteurized cow’s milk (CM; 3.12% fat, 3.45% protein, 4.57% lactose, pH 6.70) was purchased from a local market in Kaunas, Lithuania. Commercial concentrated lyophilized starter culture Lactoferm ABT 9 for direct inoculation (DVI^®^), consisting of a combination of *Streptococcus thermophilus* and probiotic-type cultures of *Bifidobacterium bifidum* and *Lactobacillus acidophilus,* was supplied by Biochem (Montelibretti, Italy). The starter was stored in freeze-dried form at −22 °C until use.

### 2.2. White Kidney Bean Extract Preparation

**Figure 1:**
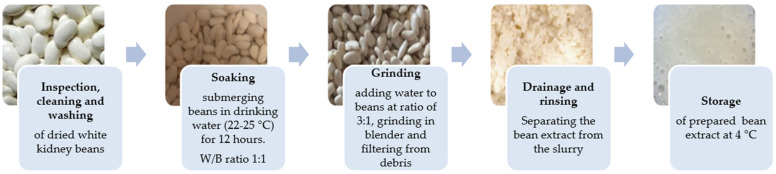
Flow chart of bean extract preparation.

The preparation of white kidney bean extract followed a research methodology modified from the process used for soy milk [11]. The flow chart of bean extract preparation is presented in Figure 1. A total of 500 g of white kidney beans were soaked overnight for 12 h in 500 L of tap water at room temperature (water-to-bean ratio of 1:1, *v*/*w*). The hydrated white kidney beans were then drained, rinsed, and ground with tap water, maintaining a water-to-soaked bean ratio of 3:1 (*v*/*w*). The soaked kidney beans were ground for 3 min using a hand blender ErgoMixx 1000 W (Bosch, Gerlingen, Germany). The resulting bean slurry was filtered through a muslin cloth to separate the insoluble residue, and the resultant extract (carbohydrates 3.53%, fats 0.16%, proteins 2.08%, ash 0.32%) was immediately used for the preparation of blends for fermentation. 

**Figure 2:**
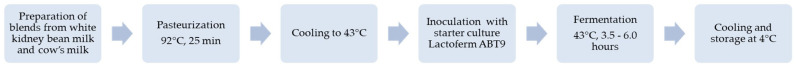
Flow chart of preparation of fermented milk-like drinks.

Three mixtures were prepared from different ratios of cow’s milk (CM) and kidney bean milk extract (BE): FMLD1 (CM 30%, BE 70%), FMLD2 (CM 50%, BE 50%), FMLD3 (CM 70%, BE 30%). These blends were compared to plain fermented kidney bean extract (FBE; CM 0%, BE 100%) and plain fermented cow’s milk (FCM; CM 100%, BE 0%) to evaluate the impact of the CM and BE ratios on the physicochemical and microbiological characteristics of the fermented drinks during refrigerated storage. Following the modified procedure of Uju et al., (2019) [11], the mixtures were subjected to pasteurization in a water bath at 92 °C for 25 min. After cooling to 43 °C, the samples were inoculated with probiotic starter culture and incubated until a pH of 4.4–4.5 was reached, then stored at 4 °C for further analysis (Figure 2). Fermented drink composition, sensory properties, amino acid profile, and microstructure were determined on day 1. During storage, on days 1, 7, 14, 21, and 28, physicochemical parameters, microbiological profile, and overall acceptability were evaluated. The experiment was repeated twice on two different dates, with tests performed three times from two biological replicates.

### 2.3. Determination of Composition

The fat, carbohydrates, nitrogen, and protein contents in the fermented samples were determined following established methods, such as 92/608/EEC:1992 [12], ISO 22184:2021 [13], ISO 8968-3:2004 [14], and the ash content was determined for the FBE using the gravimetric method with a muffle furnace at 550 °C, as described in FBE AOAC (2000) [15].

### 2.4. Determination of Amino Acids Profile

The amino acid (AA) compositions of the samples were analyzed according to ISO 13903:2005 [16], with some modifications, via ultrafast liquid chromatography (UFLC) with automated o-phthalaldehyde (OPA)/9-fluorenylmethyl chloroformate (FMOC)/Mercaptopropionic Acid (MERC) derivatization. Standard solutions of the amino acids, including alanine, aspartic acid, arginine, cystine, glycine, valine, leucine, isoleucine, threonine, serine, proline, methionine, glutamic acid, phenylalanine, lysine, histidine, tyrosine, asparagine, and tryptophan were used for this analysis (A9781 Sigma-Aldrich, Darmstadt, Germany).

To commence the analysis, each sample (approx. 0.4 g) underwent hydrolysis with 25 mL of 6 M HCl for 24 h at 103 °C. The resultant contents were quantitatively transferred into a 250 mL beaker using a 150–200 mL solution of 0.2 mol Na^+^/L, with pH 2.20, containing trisodium citrate dihydrate. The resulting hydrolysate was partially neutralized FBE through the gradual addition of 17 mL of 7.5 N sodium hydroxide solution while stirring continuously, ensuring the temperature remained below 40 °C (in a cold-water bath). The pH was adjusted to 2.20 at room temperature using a sodium hydroxide solution (7.5 N). Before injection, all samples were filtered through 0.45 μm filters. The amino acids were separated using a UHPLC column YMC-Triart C18 (1.9 μm, YMC Co. Ltd., Kyoto, Tokyo) on a UFLC instrument (Shimadzu, Japan), which was equipped with a fluorescence detector RF-20Axs and a pre-treatment function equipped with an automatic injector SIL-30AC (Shimadzu, Kyoto, Japan). The analytical conditions were as follows: mobile phase consisting of solvent A (20 mmol/L potassium phosphate buffer, pH 6.5) and solvent B (45/40/15 acetonitrile/methanol/water); flow rate, set at 0.5 mL/min; column temperature, maintained at 45 °C; detection wavelengths: RF-20Axs Ex. at 350 nm, Em. at 450 nm; Ex. at 266 nm, Em. at 305 nm (9.0 min). A calibration set comprising five levels was utilized, covering a concentration range of 9.375–150.00 μmol/L, with the exception of cysteine, covering a concentration range of 8.08–75.00 μmol/L.

### 2.5. Determination of pH and Lactic Acid Content

The pH was measured using a portable pH meter (Sartorius Professional meter for pH Measurement, Göttingen, Germany), inserting the electrode directly into the fermented sample. Three measurements for each sample from two biological replicates were taken at room temperature.

The percentage of lactic acid in yoghurt samples was determined according to ISO 11869:2012 [17]. 

### 2.6. Color Determination

The color values of samples at 1 and 28 days of storage were determined using a portable color meter PCE-CSM5 (PCE instruments, UK) following the standard measuring option CIE L* a* b* C* H*. L* refers to lightness, a* to redness/greenness, b* to yellowness/blueness, h* (hue angle) refers to the degree of the dominant spectral (red, green, or blue) component, and the c* (chroma) represents the saturation of a color. Calibration before measurement was performed with white standard plate. Color measurements were taken using the reflectance specular equipped with the D65 illuminant, a 10° observer angle, and an 8 mm aperture. Each stirred sample (40 mL) was placed in a Petri dish (diameter 90 mm), and 3 measurements were taken at 3 s intervals at approximately 20 °C. Later, the measurements were averaged for each sample. The total color difference (∆E) during sample storage was calculated as follows [18]:

∆E = [(*L* − *L_0_*)^2^ + (*a* − *a_0_*)^2^ + *(b − b_0_*)^2^]^1/2^ (1)

where L_0_, a_0_, and b_0_ are values of day 1 and *L*, *a*, and *b* are the values measured throughout the storage period.

### 2.7. Determination of Rheological Properties

Viscosity measurements were carried out using a rotational rheometer RheolabQC (Anton Paar, Graz, Germany) equipped with a flexible cup holder and a probe type CC17 according to Hanley et al., (2024) [19], with some modifications. Before measurements, the rheometer was adjusted according to the manufacturer’s instructions. Fermented samples, not stirred, in 100 mL beakers, were taken directly from the refrigerator, which maintained a 4 ± 2 °C temperature. Measurements were repeated in duplicate (with a new sample beaker taken from the refrigerator each time) at room temperature (20 ± 2 °C). The shear rate was increased from 1/s to 100/s, measuring a point every 2 s for 200 s. Data were obtained using RheoCompass Software (Version 1.12.423-Release). Apparent viscosity and shear rate data were fitted with the power law equation:

*η* = *K* (*γ̇*) *n* (2)

where *η* is apparent viscosity (Pa·s), *K* is the consistency index (Pa·sn^−1^), *γ̇* is the shear rate (s^−1^), and *n* is the flow behavior index.

### 2.8. Determination of Sensory Profile

A Quantitative Descriptive Analysis (QDA) was conducted to evaluate the sensory attributes of the samples [20]. This study adhered to ethical guidelines, having obtained approval from the Research ethics committee of the Lithuanian University of Health Sciences, and informed consent was obtained from the participants prior to this study. An 8-member semi-trained panel was selected based on specific criteria, including age range (e.g., 25–45 years), no known sensory impairments, and previous experience in sensory evaluation. Prior to this study, panelists underwent re-training over three sessions to familiarize themselves with the sensory evaluation process, specifically focusing on using a 1–10 intensity scale for assessing sensory characteristics. Training included introduction to sensory evaluation, descriptor familiarization, and practical exercises, where panelists evaluated standard reference samples to calibrate their sensory perceptions. During the training sessions, panelists engaged in structured open discussions to generate and refine the descriptors that were important for the descriptive evaluation of fermented milk-like drinks. A total of eight attributes were identified through this process: acidic odor, sourness, milk taste, aftertaste, off-flavor, firmness, transparency, and overall acceptability. The sensory evaluation was conducted in a controlled sensory laboratory, designed to minimize external influences. The room environment was maintained at a temperature of 20 °C with standardized lighting to a bright white light. Samples were presented in random order, each identified with a 3-digit random number. Panelists assessed five yoghurt samples per session, rating the intensity of each identified attribute using a 1–10 intensity scale, where 1 represented “not detectable” and 10 represented “extremely intense”. Following the QDA evaluation, statistical analyses were performed to validate the panel’s discrimination capacity, repeatability, and reproducibility. The analysis included discrimination tests to assess the panel’s ability to differentiate between samples, and repeatability and reproducibility assessments were conducted over two tasting sessions as replicates to ensure consistency in the ratings provided for the FBE by the panelists. 

### 2.9. Assessment of Microbiological Profile

For microbiological analysis of fermented drinks, samples of 1 mL were taken on refrigerated storage days 1, 7, 14, 21, and 28, diluted in 9 mL of sterile sodium chloride physiological solution (Sigma, Malmö, Sweden), mixed and submitted to 10 decimal serial dilutions. Counts of *Streptococcus thermophilus* (ST) were enumerated using the pour plate technique after aerobic incubation at 37 °C for 72 h on M17 agar (Oxoid, Wade Road Basingstoke Hampshire RG24 8PW, UK), supplemented with 10% (*w*/*v*) lactose solution (Sigma-Aldrich, Amsterdam, The Netherlands) [21]. *Bifidobacterium bifidum* (BB) counts were enumerated on TOS-propionate agar (Sigma-Aldrich, Bangalore, India) supplemented with Microbiology MUP Selective Supplement (Merck, Darmstadt, Germany) after anaerobic incubation at 37 °C for 72 h [22]. *Lactobacillus acidophilus* (LA) was enumerated on MRS agar (Sigma-Aldrich, Schaffhausen, Switzerland), supplemented with 0.1 mg/L of clindamycin and 10 mg/L ciprofloxacin, after anaerobic incubation at 37 °C for 72 h [23]. Anaerobic conditions were obtained using an AnaeroGen system (Oxoid, Tokyo, Japan). Enterobacteria were enumerated after 24 h at 37 °C on Violet Red Bile Glucose Agar (Oxoid, Wade Road Basingstoke Hampshire RG24 8PW, UK) [24]. Coliforms were enumerated on Violet Red Bile Agar (Sigma-Aldrich, Bangalore, India) after 24 h at 37 °C [25]. Yeast and molds were enumerated after 5 days at 25 °C on potato dextrose agar (Oxoid, Wade Road Basingstoke Hampshire RG24 8PW, UK) acidified with sterile 10% lactic acid solution supplement (Liofilchem, Roseto degli Abruzzi, Italy) for bacteria growth inhibition [26].

### 2.10. Determination of Microstructure 

Microstructural analysis followed the procedure outlined by [27], with some adjustments. In brief, the microstructural analysis of samples was conducted using a “Mira3” scanning electron microscope (SEM, Tescan Orsay Holding, a.s., Brno-Kohoutovice, Czech Republic). Fermented drinks were lyophilized using a freeze-dryer, mounted on a 51 mm diameter silicon wafer (MicrotoNano, Haarlem, the Netherlands) without double-sided adhesive carbon discs. Silicon was selected as the substrate due to the prevalence of CHON elements in organic samples, minimizing the elements that could skew the results and enhance the signal-to-noise ratio. The SEM operated in high vacuum mode utilizing backscattered electron (BSE) and secondary electron (SE) detectors. Magnification was increased to 1.0 kx for precise dimensional measurements and element composition analysis at 5 kV acceleration voltage. 

### 2.11. Statistical Analysis

The data processing and analysis were conducted using the SPSS statistical package (SPSS Inc., version 24, Chicago, IL, USA). Descriptive statistics (Explore) and two-way analysis of variance (ANOVA) were employed for data analysis. Differences between the means were evaluated using the Tukey test. Statistical analysis was performed at a significance level of 95%.

## 3. Results

### 3.1. Composition

Table 1 presents data on the nutritional composition of the bean extract (BE) and various types of yoghurt, with a specific focus on their carbohydrate, fat, protein, and ash content, each expressed as a percentage. All yoghurt samples (except for FCMFMLD2 and FMLD3 proteins) differed significantly in their composition, with most significant compositional differences being between the bean (FBE) and cow’s milk (FCM) yoghurt. The FBE contained the lowest carbohydrate (2.14%), fat (0.11%), protein (1.45%), and ash (0.24%) content compared to cow’s milk yoghurt (4.63%, 2.84%, 3.32%, 0.71%, respectively), with the hybrid yoghurts (FMLD1-3) showing intermediate values. 

### 3.2. Amino Acid Profile

Table 2 presents the amino acid composition in fermented drinks. A total of 17 amino acids were found in all samples, with asparagine and tryptophan being absent. Glutamic acid stood out as the predominant amino acid, comprising 18–22% of the overall amino acid composition in all samples. Most amino acids showed an increasing trend from FBE to FCM, indicating enhanced nutritional content with the increasing amounts of cow’s milk in blends. Significantly higher amounts of glutamic acid, serine, histidine, threonine, alanine, tyrosine, cystine, valine, methionine, phenylalanine, isoleucine, leucine, and lysine were found in the FCM compared to the FBE. Some amino acids, such as glycine and arginine, remained relatively consistent across all samples. Proline was below detection levels in the fermented bean extract and in some blends (FMLD1 and FMLD2). The amino acid composition in the samples varied based on the ratio of bean extract to cow’s milk used, with the fermented cow’s milk demonstrating significantly higher content of most essential amino acids compared to fermented bean extract. The fermented blends exhibited intermediate levels of amino acids, with the trend increasing towards higher cow’s milk ratios.

### 3.3. pH and Lactic Acid Content

On day 1, the pH values of cow’s milk yoghurt (FCM), at 4.57, and those of the blended samples (FMLD1–3) were similar (Table 3), averaging around 4.50, and were significantly lower than the pH of the bean extract (FBE), at 4.64. All samples then experienced a drop in pH from day 1 to day 7, followed by an increase by day 14. The final pH values of the fermented cow’s milk and blends were generally lower than their initial pH on day 1 (*p* ≤ 0.05). 

Table 4 depicts the lactic acid changes in fermented drinks during 28-day storage. The lactic acid production was influenced by the ratio of bean to cow’s milk used in the formulation. The FBE demonstrated significantly lower lactic acid content compared to the remaining samples throughout all storage periods (*p* ≤ 0.05). FMLD3 and the FCM had the highest lactic acid content compared to the other samples during the entire storage duration (*p* ≤ 0.05).

### 3.4. Colour

Our study found that drinks made from cow’s milk (FCM) and blends (FMLD1–3) exhibited a whiter and more yellow color (higher L* and b* values, *p* ≤ 0.05) compared to the fermented kidney bean extract (FBE), which had a bluish-greenish tint (higher a* value, *p* ≤ 0.05) on the first day of analysis (Table 5). Furthermore, plain FCM and the blend containing 70% cow’s milk (FMLD3) had significantly higher color purity, as indicated by the highest values of c*. The bluish-greenish tint can be observed in the fermented kidney bean extract (h* (hue angle) values). 

The hue angle (h*) values confirmed that on day 1, the dominant spectral component in the FBE was green, while the remaining samples, especially plain FCM, were more yellowish in color. This indicates that the addition of cow’s milk to the blends (FMLD1–3) resulted in a shift towards a more yellowish hue compared to the greenish hue observed in the fermented bean extract. 

**Figure 3:**
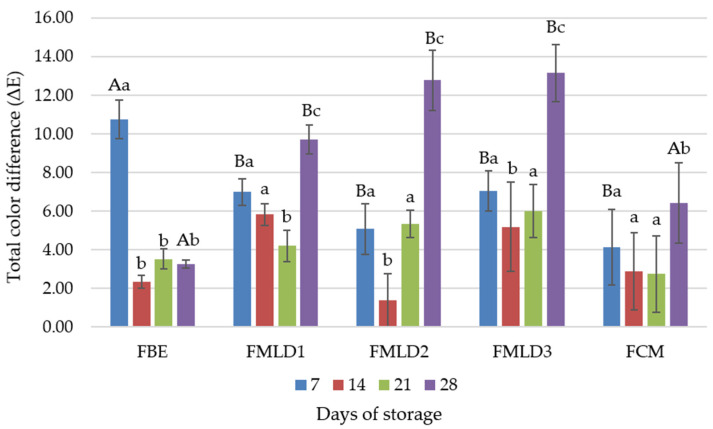
Total color difference in yoghurt during 28 days of storage. Values labelled with different uppercase letters indicate significant (*p* ≤ 0.05) differences between samples, while values labelled with different lowercase letters indicate significant (*p* ≤ 0.05) differences between storing days. FMLD1 (30% cow’s milk and 70% white kidney bean extract), FMLD2 (50% cow’s milk and 50% white kidney bean extract), FMLD3 (70% cow’s milk and 30% white kidney bean extract), FBE (100% white kidney bean extract), FCM (100% cow’s milk).

All color coordinates changed unevenly during the 28-day storage period of the fermented drinks. However, on day 7, the highest color difference (Figure 3) was observed in the FBE, while the samples containing cow’s milk (FMLD1–3 and FCM) had significantly (*p* ≤ 0.05) fewer color changes. By day 28, the most significant color changes were found in blended samples, while the plain samples (FBE and FCM) showed fewer (*p* ≤ 0.05) changes.

### 3.5. Rheological Properties

**Figure 4:**
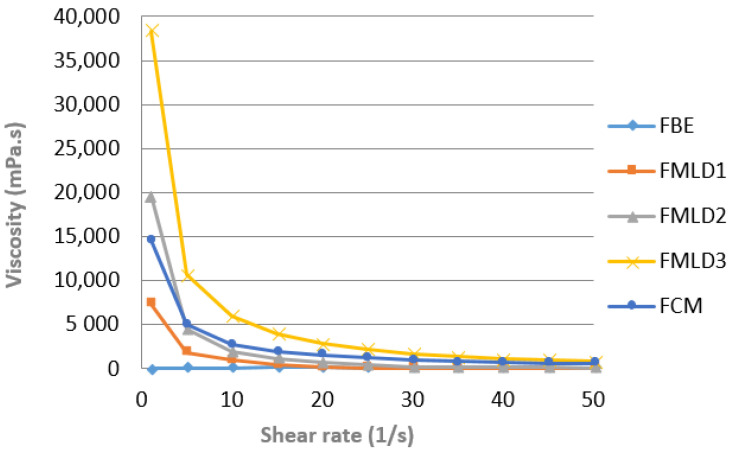
Flow curves (apparent viscosity and share rate relationship) of fermented drinks on day 1. FMLD1 (30% cow’s milk and 70% white kidney bean extract), FMLD2 (50% cow’s milk and 50% white kidney bean extract), FMLD3 (70% cow’s milk and 30% white kidney bean extract), FBE (100% white kidney bean extract), FCM (100% cow’s milk).

**Figure 5:**
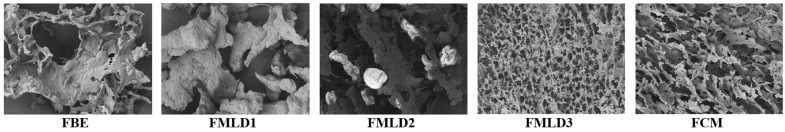
Scanning electron micrographs of the samples (SEM HV: 5.0 kV. SEM MAG: 1.00 kx., bar = 50 µm). FMLD1 (30% cow’s milk and 70% white kidney bean extract), FMLD2 (50% cow’s milk and 50% white kidney bean extract), FMLD3 (70% cow’s milk and 30% white kidney bean extract), FBE (100% white kidney bean extract), FCM (100% cow’s milk).

The flow curves of the samples at shear rate 0–50/s on day 1 are presented in Figure 4. All samples containing cow’s milk (MLD1–3 and FCM) exhibited shear-thinning behavior (viscosity decreased with increasing shear rate); meanwhile, the FBE behaved similar to Newtonian fluids, as the increased share rate did not affect the viscosity. The fermented bean extract (FBE) had the lowest viscosity (average 66,26 mPa.s, at a shear range of 0.1–100/s) on day 1. On the other hand, the viscosity of the remaining samples, in most cases, increased as the percentage of cow’s milk in the sample increased and was, on average, the following: 546.18 mPa.s, 1421.29 mPa.s, and 3553.54 mPa.s (at a shear range of 0.1–100/s) for all blended samples (FMLD1–3). A similar trend, i.e., the lowest viscosity in FBE between samples was observed across all study samples; therefore, Figure 5 presents only day 1.

Table 6 presents the average values of the shear stress (Pa) applied to fermented drinks at the shear range of 0.1–100/s during the 28-day storage. FMLD3 consistently had the highest firmness values across all days, followed by the FCM. The FBE and FMLD1 had the lowest firmness, while FMLD2 showed low firmness at the beginning of the storage period, with significant increases at the end of it. 

### 3.6. Sensory Profile

The sensory profile of fermented drinks is presented in Table 7. 

The acidic odor was similar in all samples. The sourest taste, which was 3.06 times higher (*p* ≤ 0.05) than the sour tase of the FBE, was found in FMLD1. Meanwhile, the remaining samples did not differ significantly from each other. As expected, the most intense (*p* ≤ 0.05) milk taste was felt in the FCM and the blends (FMLD1–2), compared to the FBE and FMLD1. The weakest (*p* ≤ 0.05) aftertaste and off-flavor were expressed in the FCM. Significantly lower firmness was found in the FBE and FMLD1. The transparency value was 3.33 times higher (*p* ≤ 0.05) in the FBE compared to the FCM, while the remaining samples occupied an intermediate position and did not differ significantly.

Initially, increasing the ratio of cow’s milk in the blends significantly increased their acceptability, i.e., the fermented cow’s milk (FCM) and the blends having a considerable amount (50–70%) of cow’s milk (FMLD2 and FMLD3, respectively) were 1.77–2.02 times more acceptable compared to the plain FBE (*p* ≤ 0.05, Table 8). Interestingly, the plain fermented bean extract (FBE) and its blend FMLD1 improved in acceptability during storage, reaching the level of acceptability of the other blends and FCM. 

### 3.7. Microbiological Profile

Table 9 shows microbial viability of *L. acidophilus*, *S. thermophilus*, and *B. bifidum* in fermented drinks over a storage period of 28 days. All strains exhibited satisfactory survival counts. Comparatively, the yoghurt samples containing 50% (FMLD2), 70% (FMLD3), and 100% (FCM) of cow’s milk showed higher counts of *S. thermophilus*, reaching up to 10.18 log_10_ CFU/g in FMLD3 at day 21. Additionally, higher *L. acidophilus* numbers were determined in the FCM during the 28-day period in comparison to the samples containing fermented bean extract (*p* ≤ 0.05). However, the count of *B. bifidum* in the FBE was significantly higher than that in the FCM, with counts of 4.9 log_10_ CFU/g and 4.3 log_10_ CFU/g in the FBE and FMLD1, respectively, on day 28, in comparison to no growth in FMLD2, FMLD3, and FCM on the same day (*p* ≤ 0.05). 

All samples demonstrated the absence of Coliforms, Enterobacteria, yeasts, and molds during the 28-day period.

### 3.8. Microstructure 

Figure 5 shows the microstructural differences among the samples, as visualized by scanning electron microscopy. The fermented bean extract (FBE) expressed a less dense protein network and a grainier texture compared to the fermented cow’s milk’s (FCM) smooth texture, with a dense protein network and abundant fat globules. The blended samples (FMLD1-3) demonstrated varying degrees of intermediate characteristics, with increasing bean extract content leading to less dense protein networks, while higher cow’s milk content results in creamier textures and more abundant fat globules. The sample containing FMLD3 was characterized by a very compact structure, comprising a network of casein micelles and fat. 

## 4. Discussion

### 4.1. Composition

The nutritional value of fermented been extract (FBE) was significantly lower than that of cow’s milk (FCM), containing fewer amounts of carbohydrates, proteins, and especially fats. The samples prepared with milk blends occupied intermediate positions, according to their composition.

There is a lack of scientific literature on fermented kidney bean extract, so only white kidney bean extract (BE) compositions can be compared among research data. The BE composition in this study is similar to that described by Aydar et al. (2023) [28], where red kidney bean extract had 2.32 ± 0.20% protein, 0.4 ± 0.0% fat, and 3.00 ± 0.02% total solids, and by Zahir et al. (2024) [29], noting that the white kidney bean extract contained 2.62% protein, 0.29% fat, and 0.92% ash. Meghrabi et al. (2023) [30] detected much higher nutrient levels (carbohydrate—6.41%; fat—0.79%, protein—3.77%, and ash—1.20%) in white kidney bean extract from beans grown in Jordan. These differences in composition may appear due to different preparation methods, the growth of bean seeds, climatic conditions, etc. According to Aydar et al. (2023) [28], plant-based milk-like extracts tend to be lower in fat, making them a good option for those seeking lower-fat alternatives. Additionally, plant-based extracts contain dietary fiber, though they are generally lower in protein (except for soy milk) [31].

LAB fermentation affected the primary bean extract (BE) composition, reducing carbohydrates and proteins. The reduction of nutrients is primarily due to microorganisms utilizing carbohydrates as a carbon source to produce acids that lower the pH [29]. LAB can also produce proteolytic enzymes because they require many free amino acids to grow. The proteolytic abilities of different LAB strains can vary highly [32]. Emkani et al. (2021) [33] found proteolysis in pea extract after fermentation with *Streptococcus thermophilus*, *Lactobacillus acidophilus*, and *Bifidobacterium lactis* alone or in co-culture.

Many plant-based fermented dairy drink alternatives are naturally low in fat and carbohydrates, making them attractive options for individuals seeking to manage their weight or dietary intake [25,26]. However, studies indicate that such yoghurt alternatives may vary in their nutritional profiles. While marketed plant-based milk alternatives can be lower in fat and carbohydrates compared to cow’s milk, some varieties, such as almond, soy, oat, and coconut can actually be richer in these macronutrients, with the specific nutritional profile depending on the type of plant-based milk alternative [34]. In this regard, kidney bean extract may be beneficial for individuals following low-carb diets or managing blood sugar levels and watching their weight and cholesterol levels, as it is naturally low in fat and carbohydrates, while providing a plant-based alternative to fermented dairy drinks [35].

In order to improve the nutritional profiles of bean yoghurts, they can be produced from blends of different plant-based milk-like drinks to compensate for individual deficiencies and create nutritional profiles more similar to fermented dairy drinks [36]. 

### 4.2. Amino Acids

This study found that the protein quality related to amino acids varies among legume species [37]. Glutamic acid, aspartic acid, and leucine were the predominant amino acids in fermented bean extract (FBE) and its blends. Liang et al. (2022) [38] found a similar trend in a mung bean milk-like drink after 12 h of fermentation, while Margier et al. (2018) [39] identified the same predominant amino acids in boiled and canned beans, and Wu et al. (2021) [40] in HCl-hydrolyzed kidney beans. According to Boeck et al. (2021) [41], legume protein is relatively high in lysine, while the limiting amino acids are either tryptophan or the sulfur-containing amino acids methionine and cysteine. The results of this study partially agree with Boeck et al.‘s statement. Asparagine and tryptophan were not detected among the 17 amino acids found in all samples, including FBE. Additionally, the levels of methionine and cysteine were 2.75–3.66 times lower in the fermented bean extract compared to the fermented cow’s milk. However, the level of lysine was low in the FBE compared to FCM, which contradicts Boeck et al.‘s finding, that legume protein is relatively high in lysine. This discrepancy may be due to differences in the specific legume species used or variations in the processing methods employed.

The lack or absence of some amino acids in fermented drinks can be attributed not only to the primary composition of the raw material but also to its fermentation. It is known that lactic acid bacteria (LAB) can transform amino acids into other compounds during fermentation. Some strains can convert asparagine to aspartate and ammonia through deamidation, while others utilize proline as a nitrogen source, reducing its availability in the final product. Tryptophan can be metabolized by some bacteria into indole and other compounds, thus reducing its concentration [42]. Ziadi et al. (2008) [43] showed that *L. lactis* strains SLT6 and SLT10 produced 35 volatile compounds, including aldehydes, potent aroma compounds derived from amino acid catabolism, during growth in milk. Later, Ziadi et al. (2010) [44] found that the *L. lactis* strain SLT6 degraded leucine at a rate twice that of phenylalanine, while the strain SLT10 similarly degraded both amino acids. It is evident that the LAB metabolize certain amino acids differently. In addition, some LAB need specific nutrients, including amino acids, to grow in media. According to Meng et al. (2020) [45], the nutrients required by *L. acidophilus* LA-5 were asparagine, aspartic acid, cysteine, leucine, methionine, riboflavin, guanine, uracil, and Mn2+, and when they were added to milk, the fermentation time of fermented milk prepared by *L. acidophilus* LA-5 alone was shortened by 9 h.

To fully understand the protein quality and amino acid profile of fermented bean extracts, further research is needed, focusing on a wider range of legume species and incorporating different processing techniques. This will help elucidate the complex relationships between legume protein composition, processing, and the resulting nutritional properties of plant-based fermented drink alternatives.

### 4.3. Acidity

The pH measurement is a critical quality control step in fermented drink production. It allows for the evaluation of acid formation during fermentation, which is essential for determining the completion of the process [46]. 

The pH decreased mostly during the first 7 days of storage in all samples. LAB activity is greatly related to the availability of nutrients, pH changes, and the production of various metabolic compounds in the fermentable matrix [47]. Furthermore, the lowest pH change from day 1 to day 7 was in the fermented bean extract (FBE) (0.06) and the highest in the fermented cow’s milk (FCM) (0.24). Again, these results suggest greater lactose than polysaccharide degradation by LAB during fermentation.

In fermented cow’s milk and its blends, the pH increased on day 14 and then slightly decreased by day 28 during storage at 4 °C. This pH drop at the end of storage could be attributed to the activity of the starter rather than that of the probiotic-type strains, as we observed a significant increase in *St. thermophilus* counts in the mentioned samples. Similar acidification and an increase in the *St. thermophilus* count at the end of the storage period have been reported by Sah et al. (2015) [48]. Probiotic-type strains may stimulate the growth of starters in fermented drinks, while the kidney bean extract matrix may have a different effect on the growth of strains. Wang, Yu, Yang, and Chou (2003) [49] also noted lower pH values in soy extract fermented with mixed cultures of *Lactobacillus acidophilus* and bifidobacteria compared to the extract fermented with *L. acidophilus* alone after 24 h of fermentation. These observations align with the viable count results described in our study (Table 9). The selected probiotic-type strains might be particularly well-adapted to the substrates present in the bean extract. Their enzymatic systems can be more efficient in breaking down proteins, fats, and carbohydrates [50], providing rapid acidification in 3.0–3.5 h during fermentation, which was observed in the bean extract-containing fermented drinks (FBE, FMLD1-2). Thus, the different dynamics of pH fluctuation from day 14 in both the plain samples and their blends could be attributed to the variability in survival of starter strains and the formation of varying amounts of alkaline protein breakdown products under the action of their proteolytic enzymes. 

Lactic acid plays a crucial role in determining the structure, texture, and sensory attributes of fermented drinks. The observed changes in lactic acid content showed the higher initial levels in lactic acid percentage in mainly cow’s milk-containing samples (particularly FCM, avg. 0.77%) compared to the plain fermented bean extract (FBE, 0.19%). This is in alignment with findings in similar studies examining the fermentation process and acid production in dairy products with plant-based additives [51], where none of the commercial plant-based fermented drinks (soy, coconut, cashew, almond, and hemp) had such a high concentration of lactic acid as the dairy yoghurt did (avg. 0.31 vs. 1.11, respectively). LAB ferment mainly hexoses (glucose, fructose, galactose, mannose), disaccharides (lactose, maltose, sucrose), and some of them also ferment pentoses [50]. Since the dominant carbohydrates in bean seeds are polysaccharides [52], the absence of simple sugars leads to lower lactic acid formation [53], which impacts the overall structure, texture, and sensory attributes of the final product, in comparison to dairy-based fermented drinks. The initial rise in lactic acid, especially in samples with plant-based additives (FMLD1-3), is consistent with an enhanced fermentation activity due to additional substrates for LAB. The stabilization and slight fluctuations in lactic acid levels over time reflect the dynamic balance between LAB activity and acid consumption or neutralization processes, commonly observed in fermentation studies [31].

### 4.4. Color

The appearance and color of fermented drink can influence consumer acceptability [54]. The fermented cow’s milk (FCM) and the blends (MLD1-3) exhibited a whiter and yellower (*p* ≤ 0.05) color compared to the fermented kidney bean extract (FBE), which displayed a bluish-greenish tint in this study. In addition, the FCM and the blends with up to 70% cow’s milk (FMLD3) had a significantly higher purity of color. Since scientists have studied soybeans and soy-based products the most, there is very limited data available on other fermented bean extract products. However, the results of this study are partially in agreement with Grasso et al. (2020) [51], where plant-based (coconut, almond, hemp) yoghurts were darker and less yellow than cow’s milk yoghurt. Marlapati et al. (2024) [55] also found that dairy yoghurt was significantly whiter than coconut, oat and almond yoghurt, while other color coordinates varied between samples. The FBE in this study was darker (L* 37.41 vs. 70.07), greener (a* −0.72 vs. 2.82), and less yellow (b* 0.81 vs. 20.09) than the yoghurt-type bean-based beverages in Cichonska et al.’s (2022) study [56]. This can be explained by the relationship between the sizes of protein and fat globules in yoghurt, and their ability to scatter and reflect light, which is directly connected to the overall brightness of yoghurts [51]. The larger protein and fat globules in fermented cow’s milk and blends tend to scatter and reflect light more effectively, resulting in a whiter and more opaque appearance. Conversely, smaller globules may allow more light to pass through, leading to a more translucent or bluish-greenish tint, as observed in the fermented kidney bean extract (h* (hue angle) values). A greenish-greyish color, associated with various types of plant-based milk alternatives, was also reported by Tangyu et al. (2019) [57]. 

All color coordinates changed unevenly during the 28 days of storage; nevertheless, on day 7, the highest (*p* ≤ 0.05) color difference was observed in FBE. It has been stated that LAB ferment lactose and selectively degrade casein in cow’s milk [58] but have limited ability to ferment polysaccharides such as starch or dextrin, since they lack the necessary hydrolytic enzymes [59]. The carbohydrates in white bean seeds contain approximately 40 g of starch and 20 g of dietary fiber per 100 g [52], while lactose is absent. Thus, bifidobacteria fermentation of different substrates significantly lowers the percentage of lactic acid, and possibly unusual by-products of fermentation in the FBE could affect not only the color but also the taste and rheological parameters [52,53].

### 4.5. Viscosity 

Viscosity is often used to determine a product’s “mouthfeel” because it is a key factor in our perception and appreciation of food products [60]. In our study, all samples containing cow’s milk (FMLD1-3 and FCM) exhibited shear-thinning behavior, which means that their viscosity decreased as the shear rate increased. This characteristic is common in dairy products, making them easier to pour and mix, which enhances mouthfeel and consumer satisfaction. In contrast, the FBE behaved as a Newtonian fluid, maintaining constant viscosity regardless of shear rate, suggesting that its internal structure does not change under stress, affecting its texture, as it may not flow as easily as the fermented cow’s milk. On day 1, the FBE had the lowest viscosity at 66.26 mPa·s, contributing to a thinner texture compared to the FCM, which had higher viscosities, ranging from 546.18 mPa·s to 3553.54 mPa·s, indicating that the protein and fat content in fermented cow’s milk significantly enhance the gelation process and thickening properties [58]. In fact, during rheology measurements, the FBE behaved similar to unfermented soy milk in Li et al.’s (2014) [61] study. These differences in viscosity and flow behavior have important implications for product formulation and consumer acceptance; the creamy texture of fermented cow’s milk may appeal more to consumers. The lower viscosity of fermented kidney bean extract and that of the blends with a high ratio of bean extract may affect marketability if sold as set yogurt; therefore, for better positioning in the market, a specific name, such as “fermented drink” should be clearly stated on the products’ label, along with packaging that corresponds to the drinkability of the novel product. To mimic the mouthfeel and structure provided by native or acid-gelled protein in bovine products, authors developing plant-based yoghurts frequently use fructooligosaccharides, maltodextrin, pectin, and inulin as bulking agents, sucrose and granulated sugar as an extra carbon source, or gellan and locust bean gum as thickeners [26,58,59,62]. Combinations of these additives are used to obtain a desirable texture, either directly or through polysaccharide–protein interactions [63]. Kosterina et al. (2020) [64] found that adding a coconut milk alternative to yoghurt increased its viscosity due to thixotropic behavior and thicker textural consistency, which aligns with our presented data, showing that an increasing ratio of cow’s milk in the samples resulted in the highest maximum stress values, indicating greater resistance to shear forces and firmness.

Carbohydrate metabolism produces lactic acid, which acidifies products and is crucial for the development of flavor and texture, especially in fermented dairy drinks such as yoghurts [31], enabling more protein–protein interactions and crosslinking, thus contributing to increased viscosity in legume extracts [57]. In addition, it is known that the viscosity of fermented drinks positively correlates with the higher content of proteins in milk [65]. The FBE had a significantly lower percentage of protein (1.45 ± 0.02) and carbohydrate (2.14 ± 0.01) compared to the FCM (3.32 ± 0.02 and 4.63 ± 0.01, respectively), and no additives were used for the FBE in this study. However, the blend of bean extract (30%) and cow (70%) milk (FMLD3) showed the highest viscosity across all days, possibly suggesting synergistic reactions between proteins, carbohydrates, and LAB. This finding indicates that the optimal ratio of bean extract to cow’s milk in the formulation can result in a product with desirable viscosity and texture properties, even without the addition of thickening agents or stabilizers. 

### 4.6. Microstructure and Sensory Analysis

The sensory evaluation showed that the firmness and overall acceptability of the fermented cow’s milk (FCM) and those of the blends containing cow’s milk were rated with higher scores compared to the plain fermented bean extract (FBE). According to Meghrabi et al. (2023) [30], consumers can reject legume-based extracts due to their characteristic beany and earthy aroma, which is attributed to the presence of hexanol, resulting from plant lipid oxidation. 

Other studies have also found that plant-based yoghurts were perceived as thin, watery, sour, and having an undesirable appearance and as a result, were negatively associated with consumer liking, highlighting the importance of aligning sensory profiles with expectations [64,65,66]. The addition of salt significantly improved the acceptability of the FBE, suggesting that the right combination of spices and consistency-enhancing additives could lead to the development of an innovative niche product. While the current low viscosity and firmness warrant the classification of this product as a fermented bean-based beverage, the promising results from the sensory evaluation indicate that further optimization of the formulation could result in a high-quality fermented cow’s milk substitute. 

It is worth mentioning that the microbial activity during FBE storage broke down the precursor compounds that contribute to the ‘beany’ taste, producing new compounds that mask or neutralize the undesirable flavors, enhancing the overall flavor balance through the production of desirable aroma and taste compounds. This gradual change in the flavor profile during storage resulted in a more neutral and appealing taste, making this drink more palatable to consumers. 

The microstructure of plant-based yoghurt (FMLD3) made from 30% bean extract and 70% cow’s milk exhibited favorable characteristics, showing a denser and more compact structure compared to plain fermented cow’s milk (FCM), and leading to increased apparent viscosity and an enhanced ability to retain the whey phase. The ability of the bean extract to act as a natural thickening agent in the yoghurt samples containing 30–50% bean extract, which scored similarly to the FCM, highlights its potential as a functional ingredient in the production of yoghurt-type products. The panelists likely barely noticed the ‘beany’ taste in this yoghurt samples containing a low ratio of bean extract due to the dilution effect.

### 4.7. Microbial Viability

*Lactobacillus acidophilus* survived better in fermented cow’s milk compared to fermented bean extract. This is due to the presence of lactose, which is the preferred carbohydrate source for *L. acidophilus*, and the availability of casein and whey proteins in cow’s milk. The controlled pH and lack of inhibitory compounds in cow’s milk also create a more favorable environment for the growth and survival of *L. acidophilus*. In contrast, bean extract lacks lactose, contains different proteins, and may have inhibitory compounds. These factors do not support the same beneficial microbial interactions, leading to reduced survival of *L. acidophilus* in the FBE.

*Streptococcus thermophilus*, a common yoghurt starter culture, efficiently utilizes lactose and breaks down casein in milk, producing lactic acid and interacting symbiotically with other lactic acid bacteria. However, in the non-dairy (bean) extract, the absence of lactose and the different protein composition led to slower fermentation and reduced viability of *S. thermophilus* during storage. This is likely due to the more acidic environment and the presence of natural antimicrobial compounds in beans.

On the other hand, *Bifidobacterium bifidum* may survive better in fermented bean extract, similarly to other bean-based milk products [66]. This can be attributed to the presence of beneficial oligosaccharides and prebiotics in beans, as well as a more favorable pH and acid profile, and the potential tolerance of *Bifidobacterium* to any antimicrobial compounds in beans [62]. The presence of prebiotics in fermented bean drinks can contribute to the development of symbiotic food compositions, containing both probiotics and prebiotics, as supported by the microbiological analysis [38].

## 5. Conclusions

The current study was conducted on the composition, physicochemical properties, rheology, sensory behavior, and microbiological viability of five types of fermented drink compositions produced from white kidney bean extract, cow’s milk, and their blends in various proportions. This comparative analysis noted that the addition of bean extract to the blends had several effects, including enhancing the viscosity of blended drinks (FMLD3), decreasing the carbohydrate and fat contents (FBE, FMLD 1–3), and offering customers new probiotic-type fermented milk-like drinks that provide an ideal base for the growth of bifidobacteria. This study demonstrates the potential of using white kidney bean extract and its blends with cow’s milk to create a variety of fermented milk-like products with unique properties and health benefits. It highlights the importance of enhancing the sensory qualities of plain yogurt made from bean extract and its blends with cow’s milk. Thus, future research directions could aim to optimize the processing conditions and formulations to improve sensory perception, minimize off-flavors, and explore the use of slurry for human nutrition or animal feed production.

## Figures and Tables

**Table 1:** Nutritional composition of fermented drinks.

Drink Type	Carbohydrate, %	Fat, %	Protein, %	Ash, %
FBE	2.14 ± 0.01 ^a^	0.11 ± 0.01 ^a^	1.45 ± 0.02 ^a^	0.24 ± 0.03 ^a^
FMLD1	3.28 ± 0.01 ^b^	0.95 ± 0.06 ^b^	2.12 ± 0.03 ^b^	0.43 ± 0.03 ^b^
FMLD2	3.58 ± 0.01 ^c^	1.60 ± 0.03 ^c^	2.54 ± 0.32 ^c^	0.53 ± 0.03 ^c^
FMLD3	4.39 ± 0.01 ^d^	2.24 ± 0.03 ^d^	2.82 ± 0.04 ^c^	0.62 ± 0.02 ^d^
FCM	4.63 ± 0.01 ^e^	2.84 ± 0.10 ^e^	3.32 ± 0.02 ^d^	0.71 ± 0.03 ^e^

Values labeled with different lowercase letters indicate significant (*p* ≤ 0.05) differences between samples. FMLD1 (30% cow’s milk and 70% white kidney bean extract), FMLD2 (50% cow’s milk and 50% white kidney bean extract), FMLD3 (70% cow’s milk and 30% white kidney bean extract), FBE (100% white kidney bean extract), FCM (100% cow’s milk).

**Table 2:** Amino acid (μmol/L) composition in fermented drinks.

Amino Acid	FBE	FMLD1	FMLD2	FMLD3	FCM
Aspartic acid	0.23 ± 0.02 ^a^	0.21 ± 0.01 ^ab^	0.24 ± 0.02 ^a^	0.27 ± 0.01 ^a^	0.28 ± 0.03 ^ac^
Glutamic acid	0.28 ± 0.03 ^a^	0.38 ± 0.02 ^ab^	0.48 ± 0.03 ^b^	0.60 ± 0.02 ^c^	0.67 ± 0.08 ^c^
Serine	0.11 ± 0.01 ^a^	0.12 ± 0.01 ^a^	0.13 ± 0.01 ^ac^	0.16 ± 0.01 ^bc^	0.17 ± 0.02 ^b^
**Histidine**	0.02 ± 0.00 ^a^	0.03 ± 0.01 ^a^	0.04 ± 0.01 ^a^	0.04 ± 0.01 ^a^	0.07 ± 0.01 ^b^
Glycine	0.06 ± 0.01 ^a^	0.05 ± 0.00 ^a^	0.05 ± 0.00 ^a^	0.05 ± 0.00 ^a^	0.05 ± 0.01 ^a^
**Threonine**	0.08 ± 0.01 ^a^	0.10 ± 0.01 ^ab^	0.12 ± 0.01 ^bc^	0.14 ± 0.00 ^cb^	0.15 ± 0.02 ^c^
Arginine	0.10 ± 0.01 ^a^	0.10 ± 0.01 ^a^	0.10 ± 0.01 ^a^	0.11 ± 0.00 ^a^	0.11 ± 0.01 ^a^
Alanine	0.07 ± 0.01 ^a^	0.08 ± 0.01 ^a^	0.08 ± 0.00 ^ab^	0.10 ± 0.00 ^b^	0.10 ± 0.01 ^b^
Tyrosine	0.06 ± 0.01 ^a^	0.08 ± 0.00 ^ab^	0.10 ± 0.01 ^b^	0.13 ± 0.01 ^cb^	0.15 ± 0.02 ^c^
Cysteine	0.03 ± 0.00 ^a^	0.04 ± 0.01 ^a^	0.10 ± 0.00 ^b^	0.06 ± 0.01 ^c^	0.11 ± 0.01 ^b^
**Valine**	0.07 ± 0.01 ^a^	0.09 ± 0.01 ^ab^	0.10 ± 0.01 ^b^	0.15 ± 0.00 ^c^	0.14 ± 0.01 ^c^
**Methionine**	0.04 ± 0.01 ^a^	0.06 ± 0.01 ^ab^	0.06 ± 0.01 ^ab^	0.09 ± 0.03 ^b^	0.11 ± 0.01 ^bc^
**Phenylalanine**	0.10 ± 0.01 ^a^	0.11 ± 0.01 ^a^	0.12 ± 0.01 ^a^	0.15 ± 0.01 ^b^	0.15 ± 0.02 ^b^
**Isoleucine**	0.08 ± 0.01 ^a^	0.09 ± 0.01 ^a^	0.10 ± 0.01 ^a^	0.14 ± 0.01 ^b^	0.14 ± 0.01 ^b^
**Leucine**	0.14 ± 0.02 ^a^	0.18 ± 0.01 ^ab^	0.21 ± 0.01 ^b^	0.28 ± 0.01 ^c^	0.28 ± 0.03 ^c^
**Lysine**	0.08 ± 0.00 ^a^	0.14 ± 0.00 ^b^	0.16 ± 0.01 ^bc^	0.20 ± 0.03 ^c^	0.20 ± 0.03 ^c^
**Tryptophan**	nd	nd	nd	nd	nd
Asparagine	nd	nd	nd	nd	nd
Proline	nd	nd	nd	0.08 ± 0.03 ^a^	0.07 ± 0.00 ^a^

Values labelled with different lowercase letters within the same row indicate significant (*p* ≤ 0.05) differences between samples. Essential amino acids are presented in bold. FMLD1 (30% cow’s milk and 70% white kidney bean extract), FMLD2 (50% cow’s milk and 50% white kidney bean extract), FMLD3 (70% cow’s milk and 30% white kidney bean extract), FBE (100% white kidney bean extract), FCM (100% cow’s milk). nd—not detected.

**Table 3:** pH changes in fermented drinks during storage.

Day	FBE	FMLD1	FMLD2	FMLD3	FCM
1	4.64 ± 0.02 ^Aa^	4.54 ± 0.02 ^Ba^	4.48 ± 0.02 ^Ba^	4.49 ± 0.0 ^Ba^	4.57 ± 0.04 ^Ba^
7	4.58 ± 0.02 ^Ab^	4.22 ± 0.01 ^Bb^	4.31 ± 0.01 ^Cb^	4.20 ± 0.01 ^Bb^	4.33 ± 0.02 ^Cb^
14	4.74 ± 0.02 ^Ac^	4.37 ± 0.01 ^Ac^	5.27 ± 0.2 ^Bc^	5.72 ± 0.28 ^Cc^	4.81 ± 0.04 ^Aa^
21	4.67 ± 0.02 ^Aa^	4.32 ± 0.01 ^Bd^	4.46 ± 0.02 ^Ca^	4.35 ± 0.01 ^Ba^	4.48 ± 0.01 ^Cc^
28	4.64 ± 0.01 ^Aa^	4.38 ± 0.01 ^Bc^	4.49 ± 0.01 ^Ca^	4.34 ± 0.01 ^Da^	4.43 ± 0.01 ^Ec^

Values labelled with different uppercase letters within the same row indicate significant (*p* ≤ 0.05) differences between samples; values labelled with different lowercase letters within the same column indicate significant (*p* ≤ 0.05) differences between storing days. FMLD1 (30% cow’s milk and 70% white kidney bean extract), FMLD2 (50% cow’s milk and 50% white kidney bean extract), FMLD3 (70% cow’s milk and 30% white kidney bean extract), FBE (100% white kidney bean extract), FCM (100% cow’s milk).

**Table 4:** Lactic acid changes in fermented drinks during storage.

Day	FBE	FMLD1	FMLD2	FMLD3	FCM
1	0.21 ± 0.01 ^Aa^	0.50 ± 0.00 ^Ba^	0.57 ± 0.00 ^Ca^	0.74 ± 0.00 ^Da^	0.79 ± 0.00 ^Ea^
7	0.22 ± 0.00 ^Aa^	0.52 ± 0.00 ^Ba^	0.57 ± 0.00 ^Ca^	0.74 ± 0.00 ^Da^	0.80 ± 0.01 ^Ea^
14	0.18 ± 0.01 ^Ab^	0.39 ± 0.02 ^Bb^	0.43 ± 0.01 ^Cb^	0.75 ± 0.00 ^Da^	0.76 ± 0.00 ^Da^
21	0.18 ± 0.00 ^Ab^	0.47 ± 0.00 ^Ba^	0.54 ± 0.02 ^Cc^	0.72 ± 0.00 ^Dc^	0.78 ± 0.01 ^Ea^
28	0.16 ± 0.00 ^Ab^	0.46 ± 0.03 ^Ba^	0.50 ± 0.00 ^Bd^	0.76 ± 0.01 ^Ca^	0.74 ± 0.00 ^Cb^

Values labelled with different uppercase letters within the same row indicate significant (*p* ≤ 0.05) differences between samples; values labelled with different lowercase letters within the same column indicate significant (*p* ≤ 0.05) differences between storing days. FMLD1 (30% cow’s milk and 70% white kidney bean extract), FMLD2 (50% cow’s milk and 50% white kidney bean extract), FMLD3 (70% cow’s milk and 30% white kidney bean extract), FBE (100% white kidney bean extract), FCM (100% cow’s milk).

**Table 5:** CIELab color coordinates of fermented drinks during storage.

	Day	FBE	FMLD1	FMLD2	FMLD3	FCM
L*	1	37.41 ± 0.44 ^Aa^	48.15 ± 1.04 ^Ba^	51.15 ± 0.51 ^Ba^	53.81 ± 1.91 ^Ba^	50.73 ± 2.63 ^Ba^
	7	47.03 ± 0.36 ^Ab^	53.48 ± 0.47 ^Bb^	54.20 ± 1.15 ^Bb^	59.22 ± 1.14 ^Cb^	53.48 ± 0.40 ^Ba^
	14	39.41 ± 0.51 ^Ac^	42.33 ± 0.48 ^Bc^	50.07 ± 1.11 ^Ba^	48.70 ± 0.64 ^Bc^	48.00 ± 0.50 ^Ba^
	21	39.56 ± 0.28 ^Ac^	44.36 ± 0.13 ^Bd^	46.18 ± 0.26 ^Cc^	48.06 ± 0.75 ^Dc^	52.10 ± 0.46 ^Ea^
	28	40.53 ± 0.32 ^Ac^	38.48 ± 0.39 ^Ae^	38.46 ± 1.14 ^Ad^	40.70 ± 0.54 ^Ad^	44.37 ± 0.58 ^Bb^
a*	1	−0.72 ± 0.03 ^Aa^	−0.27 ± 0.04 ^Ba^	−0.23 ± 0.02 ^Ba^	−0.18 ± 0.14 ^Ba^	−0.16 ± 0.09 ^Ba^
	7	2.26 ± 0.43 ^Ab^	2.73 ± 0.16 ^Ab^	2.57 ± 0.74 ^Ab^	3.05 ± 0.41 ^Ab^	1.96 ± 0.26 ^Ab^
	14	−0.21 ± 0.10 ^Aa^	−0.07 ± 0.01 ^Ba^	0.23 ± 0.05 ^Ca^	0.44 ± 0.02 ^Ca^	0.36 ± 0.03 ^Cc^
	21	1.35 ± 0.09 ^Ac^	1.22 ± 0.12 ^Ac^	1.38 ± 0.05 ^Aa^	1.48 ± 0.04 ^Ac^	1.10 ± 0.09 ^Ad^
	28	0.14 ± 0.10 ^Aa^	0.39 ± 0.05 ^Ad^	0.56 ± 0.11 ^Aa^	0.36 ± 0.11 ^Aa^	0.48 ± 0.06 ^Ac^
b*	1	0.81 ± 0.23 ^Aa^	3.26 ± 0.05 ^Ba^	3.71 ± 0.11 ^Ba^	4.91 ± 0.14 ^Ba^	4.72 ± 0.54 ^Ba^
	7	4.57 ± 0.43 ^Ab^	6.61 ± 0.02 ^Bb^	6.63 ± 0.90 ^Bb^	8.03 ± 0.16 ^Cb^	6.68 ± 0.17 ^Bb^
	14	1.86 ± 0.10 ^Ac^	2.94 ± 0.14 ^Ba^	3.93 ± 0.17 ^Ca^	4.33 ± 0.06 ^Dc^	4.39 ± 0.03 ^Ea^
	21	2.63 ± 0.63 ^Ac^	4.22 ± 0.06 ^Bc^	4.70 ± 0.04 ^Ba^	5.33 ± 0.02 ^Bd^	5.07 ± 0.04 ^Ba^
	28	0.45 ± 0.25 ^Aa^	2.77 ± 0.27 ^Ba^	2.48 ± 0.18 ^Bc^	3.90 ± 0.10 ^Ce^	4.30 ± 0.28 ^Ca^
c*	1	1.19 ± 0.03 ^Aa^	3.28 ± 0.04 ^Ba^	3.72 ± 0.11 ^Ba^	4.92 ± 0.14 ^Ca^	4.72 ± 0.54 ^Ca^
	7	5.1 ± 0.58 ^Ab^	7.16 ± 0.06 ^Bb^	7.11 ± 1.10 ^Bb^	8.59 ± 0.28 ^Cb^	6.96 ± 0.23 ^Bb^
	14	1.88 ± 0.09 ^Ac^	2.95 ± 0.14 ^Ba^	3.94 ± 0.17 ^Ca^	4.35 ± 0.06 ^Dc^	4.41 ± 0.03 ^Ea^
	21	2.95 ± 0.60 ^Ad^	4.39 ± 0.03 ^Bc^	4.90 ± 0.02 ^Ba^	5.53 ± 0.02 ^Bd^	5.19 ± 0.05 ^Ba^
	28	0.49 ± 0.22 ^Ab^	2.80 ± 0.26 ^Ba^	2.55 ± 0.16 ^Ba^	3.92 ± 0.10 ^Ce^	4.32 ± 0.28 ^Ca^
h*	1	127.28 ± 2.83 ^Aa^	94.81 ± 0.80 ^Ba^	93.50 ± 0.29 ^Ba^	92.39 ± 2.05 ^Ba^	91.94 ± 0.80 ^Ba^
	7	63.80 ± 2.19 ^Ab^	67.57 ± 1.12 ^Ab^	69.09 ± 3.09 ^Ab^	69.27 ± 2.31 ^Ab^	73.65 ± 1.62 ^Ab^
	14	96.66 ± 3.43 ^Aa^	91.34 ± 0.18 ^Bc^	86.60 ± 0.81 ^Cc^	84.28 ± 0.15 ^Cc^	85.26 ± 0.33 ^Cc^
	21	62.65 ± 3.85 ^Ab^	73.65 ± 1.38 ^Bd^	73.64 ± 0.72 ^Bb^	74.47 ± 0.46 ^Bd^	77.77 ± 0.99 ^Bd^
	28	69.73 ± 17.54 ^Ab^	81.88 ± 1.71 ^Ae^	77.10 ± 3.23 ^Ab^	84.75 ± 1.50 ^Ac^	83.57 ± 0.82 ^Ac^

Values labelled with different uppercase letters within the same row indicate significant (*p* ≤ 0.05) differences between samples; values labelled with different lowercase letters within the same column indicate significant (*p* ≤ 0.05) differences between storing days. FMLD1 (30% cow’s milk and 70% white kidney bean extract), FMLD2 (50% cow’s milk and 50% white kidney bean extract), FMLD3 (70% cow’s milk and 30% white kidney bean extract), FBE (100% white kidney bean extract), FCM (100% cow’s milk). L* refers to lightness, a* to redness/greenness, b* to yellowness/blueness, h* (hue angle) refers to the degree of the dominant spectral (red, green, or blue) component, and the c* (chroma) represents the saturation of a color.

**Table 6:** Average shear stress (Pa) values applied to fermented drinks during the storage.

Day	FBE	FMLD1	FMLD2	FMLD3	FCM
1	3.00 ± 1.28 ^Aa^	3.51 ± 2.36 ^Aa^	7.99 ± 6.19 ^Ba^	42.72 ± 10.03 ^Ca^	29.11 ± 2.48 ^Da^
7	3.02 ± 1.04 ^Aa^	8.89 ± 2.68 ^Bb^	7.87 ± 7.36 ^Ba^	27.00 ± 12.12 ^Cb^	35.39 ± 5.65 ^Db^
14	1.10 ± 0.89 ^Ac^	11.13 ± 7.13 ^Bc^	38.14 ± 7.30 ^Cb^	46.62 ± 13.14 ^Da^	17.93 ± 2.66 ^Ec^
21	4.31 ± 1.37 ^Ad^	6.25 ± 2.12 ^Ad^	24.50 ± 8.59 ^Bc^	54.28 ± 10.75 ^Ca^	33.24 ± 5.04 ^Db^
28	3.19 ± 0.74 ^Aa^	4.68 ± 1.28 ^Aa^	26.94 ± 10.64 ^Bc^	50.08 ± 11.74 ^Ca^	30.24 ± 3.91 ^Da^

Values labelled with different uppercase letters within the same row indicate significant (*p* ≤ 0.05) differences between samples; values labelled with different lowercase letters within the same column indicate significant (*p* ≤ 0.05) differences between storing days. FMLD1 (30% cow’s milk and 70% white kidney bean extract), FMLD2 (50% cow’s milk and 50% white kidney bean extract), FMLD3 (70% cow’s milk and 30% white kidney bean extract), FBE (100% white kidney bean extract), FCM (100% cow’s milk).

**Table 7:** Sensory profile of fermented drinks on day 1.

Descriptors	FBE	FMLD1	FMLD2	FMLD3	FCM
Acidic odor	4.80 ± 3.56 ^a^	4.30 ± 2.36 ^a^	4.40 ± 2.77 ^a^	3.10 ± 0.65 ^a^	3.50 ± 1.22 ^a^
Sourness	2.30 ± 1.92 ^a^	7.04 ± 1.56 ^bc^	6.10 ± 1.85 ^ca^	5.30 ± 2.71 ^ca^	4.30 ± 2.25 ^ca^
Milk taste	1.60 ± 1.14 ^a^	3.70 ± 1.72 ^a^	6.90 ± 1.24 ^b^	7.80 ± 0.57 ^b^	8.30 ± 1.30 ^b^
Aftertaste	7.80 ± 2.20 ^a^	5.30 ± 2.39 ^ab^	4.30 ± 1.40 ^b^	5.40 ± 2.04 ^ba^	0.30 ± 0.45 ^c^
Off-flavor	8.90 ± 0.65 ^a^	5.66 ± 2.55 ^b^	1.70 ± 0.97 ^c^	0.90 ± 0.22 ^c^	0.40 ± 0.42 ^c^
Firmness	1.50 ± 0.87 ^a^	2.60 ± 0.42 ^a^	5.60 ± 1.64 ^b^	7.60 ± 1.52 ^b^	6.20 ± 1.44 ^b^
Transparency	5.00 ± 2.83 ^a^	3.20 ± 1.82 ^ab^	2.80 ± 2.10 ^ab^	2.10 ± 0.55 ^ab^	1.50 ± 0.71 ^b^
Overall Acceptability	4.40 ± 2.68 ^a^	6.30 ± 1.72 ^ab^	7.80 ± 1.75 ^b^	8.60 ± 0.89 ^b^	8.90 ± 0.74 ^b^

Values labelled with different lowercase letters within the same row indicate significant (*p* ≤ 0.05) differences between samples. FMLD1 (30% cow’s milk and 70% white kidney bean extract), FMLD2 (50% cow’s milk and 50% white kidney bean extract), FMLD3 (70% cow’s milk and 30% white kidney bean extract), FBE (100% white kidney bean extract), FCM (100% cow’s milk).

**Table 8:** Overall acceptability of fermented drinks during refrigerated storage.

Day	FBE	FMLD1	FMLD2	FMLD3	FCM
1	2.04 ± 2.57 ^Aa^	4.00 ± 2.32 ^Aa^	5.80 ± 2.08 ^AB^	8.16 ± 1.52 ^B^	7.90 ± 3.07 ^B^
7	0.73 ± 0.25 ^Aa^	1.67 ± 1.04 ^Ab^	4.83 ± 2.02 ^B^	7.93 ± 0.40 ^C^	9.50 ± 0.50 ^C^
14	6.88 ± 1.38 ^b^	8.75 ± 0.50 ^b^	8.25 ± 1.19	8.25 ± 1.19	8.50 ± 0.41
21	4.25 ± 2.22 ^Aa^	7.00 ± 1.35 ^Ab^	8.13 ± 1.03 ^B^	8.13 ± 0.63 ^B^	8.25 ± 0.65 ^B^
28	7.63 ± 1.25 ^b^	8.75 ± 0.65 ^b^	8.25 ± 0.65	8.38 ± 0.75	8.25 ± 1.50

Values labelled with different uppercase letters within the same row indicate significant (*p* ≤ 0.05) differences between samples; values labelled with different lowercase letters within the same column indicate significant (*p* ≤ 0.05) differences between storing days. FMLD1 (30% cow’s milk and 70% white kidney bean extract), FMLD2 (50% cow’s milk and 50% white kidney bean extract), FMLD3 (70% cow’s milk and 30% white kidney bean extract), FBE (100% white kidney bean extract), FCM (100% cow’s milk).

**Table 9:** Microbial count results of fermented drinks.

Day	FBE	FMLD1	FMLD2	FMLD3	FCM
	*S. Thermophilus*				
1	8.38 ± 0.05 ^Aa^	8.55 ± 0.02 ^Ba^	8.74 ± 0.05 ^Ba^	9.21 ± 0.10 ^Ca^	8.65 ± 0.04 ^Ba^
7	8.09 ± 0.33 ^Aa^	8.62 ± 0.28 ^Aab^	8.87 ± 0.02 ^Bab^	8.88 ± 0.09 ^Ba^	8.85 ± 0.13 ^Bab^
14	7.11 ± 0.10 ^Ab^	8.99 ± 0.12 ^Bb^	8.95 ± 0.15 ^Bb^	8.91 ± 0.34 ^Ba^	8.89 ± 0.10 ^Bb^
21	7.12 ± 0.00 ^Ab^	8.60 ± 0.11 ^Ba^	9.4 ± 0.00 ^Cc^	10.18 ± 0.00 ^Db^	9.2 ± 0.00 ^Ec^
28	7.12 ± 0.00 ^Ab^	8.61 ± 0.00 ^Bab^	9.4 ± 0.00 ^Cc^	10.18 ± 0.00 ^Db^	9.2 ± 0.00 ^Cc^
	*L. acidophilus*				
1	6.3 ± 0.00 ^a^	5.85 ± 0.47 ^Aa^	5.88 ± 0.02 ^Aa^	6.52 ± 0.12 ^Ba^	6.40 ± 0.06 ^a^
7	5.61 ± 0.01 ^Ab^	6.27 ± 0.00 ^Ba^	6.29 ± 0.05 ^Ba^	6.37 ± 0.06 ^BDa^	6.47 ± 0.03 ^Db^
14	5.45 ± 0.01 ^Ac^	6.11 ± 0.00 ^Ba^	5.88 ± 0.37 ^Aa^	6.59 ± 0.01 ^Ca^	6.60 ± 0.00 ^Cc^
21	4.48 ± 0.00 ^Ad^	6.1 ± 0.25 ^Ba^	4.9 ± 0.00 ^Ab^	6.26 ± 0.38 ^Ba^	6.69 ± 0.00 ^Cd^
28	0 ^e^	3.9 ± 0.00 ^Ab^	0 ^c^	3.95 ± 0.00 ^Ab^	4.85 ± 0.00 ^Be^
	*B. bifidus*				
1	5.88 ± 0.09 ^Aa^	5.23 ± 0.00 ^Ba^	5.27 ± 0.03 ^Ba^	5.37 ± 0.10 ^Ba^	4.94 ± 0.05 ^Ca^
7	6.17 ± 0.06 ^Ab^	5.57 ± 0.11 ^Bb^	5.33 ± 0.02 ^Ca^	5.36 ± 0.00 ^CDa^	5.18 ± 0.00 ^CEab^
14	6.08 ± 0.15 ^Aab^	5.50 ± 0.17 ^Bb^	5.32 ± 0.28 ^Ba^	5.45 ± 0.00 ^Ba^	5.24 ± 0.32 ^Bb^
21	6.76 ± 0.00 ^Ac^	6.8 ± 0.00 ^Ac^	5.74 ± 0.38 ^Ba^	6.28 ± 0.00 ^Cb^	5.54 ± 0.06 ^Bb^
28	4.9 ± 0.00 ^Ad^	4.3 ± 0.00 ^Bd^	0 ^c^	0 ^c^	0 ^c^

Values labelled with different uppercase letters within the same row indicate significant (*p* ≤ 0.05) differences between samples; values labelled with different lowercase letters within the same column indicate significant (*p* ≤ 0.05) differences between storing days. FMLD1 (30% cow’s milk and 70% white kidney bean extract), FMLD2 (50% cow’s milk and 50% white kidney bean extract), FMLD3 (70% cow’s milk and 30% white kidney bean extract), FBE (100% white kidney bean extract), FCM (100% cow’s milk).

## Data Availability

Data are contained within the article.
